# Physiological cardiotocography interpretation and neonatal morbidity: A historical pre–post cohort study in a tertiary perinatal center

**DOI:** 10.1111/aogs.70245

**Published:** 2026-05-21

**Authors:** Sophia Andres, Henning Schäffler, Kay Stankov, Annika Schmid, Mandana Khodawandi, Moritz Dimpfl, Wolfgang Janni, Beate Hüner, Frank Reister

**Affiliations:** ^1^ Department of Obstetrics and Gynecology University of Ulm Ulm Germany; ^2^ Ainovate GmbH, stat4med Frankfurt am Main Germany; ^3^ Department of Obstetrics and Gynecology University of Mannheim Mannheim Germany

**Keywords:** cardiotocography, fetal monitoring, intrapartum care, maternal morbidity, neonatal acidosis, physiological CTG interpretation

## Abstract

**Introduction:**

Cardiotocography (CTG) interpretation is prone to inter‐observer variability and may contribute to both missed fetal compromise and potentially avoidable intrapartum intervention. Physiological CTG interpretation (PCI) reframes fetal surveillance around fetal physiology and the intensity of hypoxic stress, but real‐world outcome data after unit‐wide implementation remain limited.

**Material and Methods:**

Historical pre–post cohort study with case‐mix adjustment at University Hospital Ulm, a tertiary perinatal center in Germany (~3200 births/year). We included term singleton pregnancies (≥37 + 0 weeks) with intended vaginal birth, comparing a pre‐implementation period (January 01–December 31, 2018) with a post‐implementation period after full adoption (May 01, 2022–April 30, 2023). PCI was introduced as a multicomponent implementation strategy (training, bedside facilitation, documentation changes, and sustainment). Primary outcome: composite neonatal morbidity defined as neonatal unit (NNU) transfer plus ≥1 of: umbilical artery pH <7.15, base deficit >16 mmol/L, or 5‐min Apgar score <7. Secondary outcomes: umbilical artery acid–base status, Apgar scores, NNU transfer, intrapartum interventions (e.g., oxytocin, tocolysis, fetal scalp blood sampling), mode of birth, and postpartum blood loss. Outcomes were compared using multivariable regression and propensity score matching.

**Results:**

A total of 4484 births met the inclusion criteria (2352 pre‐implementation; 2132 post‐implementation). Composite neonatal morbidity decreased from 4.10% to 2.92% (OR 0.67; 95% CI 0.434–0.958; *p* = 0.0259). Neonatal acidosis decreased (umbilical artery pH <7.10: 3.87%–2.53%; OR 0.649; 95% CI 0.450–0.919; *p* = 0.014), and NNU transfers declined (13.18%–9.19%; OR 0.667; 95% CI 0.59–0.808; *p* < 0.001). Cesarean section rates were not increased after adjustment (OR 0.880; 95% CI 0.722–1.071). Postpartum blood loss was higher post‐implementation (438 vs 497 mL; *p* < 0.001).

**Conclusions:**

Unit‐wide implementation of PCI was associated with improved neonatal outcomes, including fewer NNU admissions, without an increase in adjusted cesarean section rates. These findings support PCI as a promising framework for intrapartum fetal surveillance, warranting confirmation in multicenter studies and evaluation across different care settings.

## Graphical abstract

In this historical pre–post cohort study, the implementation of physiological cardiotocography (PCI) interpretation was associated with reduced neonatal morbidity, neonatal acidosis, and neonatal unit admission. Adjusted cesarean section rates did not increase, supporting PCI as a promising approach to intrapartum fetal surveillance.

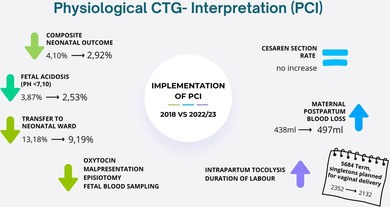

**Abbreviations**
BMI: body mass index
CI: confidence interval
CTG: cardiotocography
IQR: interquartile range
mL: milliliters
NNU: neonatal unit
OR: odds ratio
PCI: physiological CTG‐interpretation
SD: standard deviation

**Key message**

Physiological cardiotocography (CTG) interpretation uses fetal physiology to contextualize CTG changes and hypoxic and inflammatory stress. After unit‐wide implementation, neonatal acidosis and neonatal unit admissions/transfers decreased, with lower composite neonatal morbidity and no increase in adjusted cesarean section rates.

## 1 INTRODUCTION

Continuous intrapartum cardiotocography (CTG) was introduced to reduce intrapartum mortality and neonatal morbidity^1^, ^2^; however, severe hypoxia‐related neonatal complications remain challenging, and CTG interpretation continues to be associated with substantial inter‐observer variability and potentially avoidable intrapartum intervention.^3^, ^4^, ^5^, ^6^ Concurrently, caesarean and operative birth rates have increased internationally, with concern that limitations in CTG interpretation contribute to both missed fetal compromise and unnecessary interventions.^7^

Physiological CTG interpretation (PCI) has been proposed as a clinically actionable approach that reframes CTG assessment from pattern recognition and classification to physiology‐based interpretation, explicitly incorporating baseline fetal oxygenation, maternal comorbidities, and evolving intrapartum oxygen demand.^8^ This approach emphasizes early recognition of fetal compensatory responses and prioritizes reducing the intensity of ongoing hypoxic stress to the fetus (e.g., through contraction reduction and targeted use of tocolysis) rather than acceleration of labor through uterotonics when compromise is suspected.^9^, ^10^, ^11^

Recent guideline updates reflect ongoing international efforts to optimize intrapartum fetal surveillance, including NICE guideline NG229 (published December 14, 2022; updated November 12, 2025).^12^

In Germany, intrapartum fetal monitoring remains strongly influenced by FIGO‐based classification frameworks.^13^ Within this context, our unit was the first in Germany to implement PCI across the labor ward, accompanied by a structured training program and revised CTG documentation. We therefore aim:

1. To assess whether the implementation of PCI was associated with a reduction in neonatal morbidity in term births (≥37 + 0 weeks) compared with the pre‐implementation period.
2. To evaluate associations with secondary neonatal outcomes (umbilical artery acid–base status, 5‐min Apgar score, and neonatal unit [NNU] admission) and intrapartum management variables (e.g., oxytocin and tocolysis use, fetal scalp blood sampling).
3. To examine maternal and obstetric safety outcomes and balancing measures, including mode of birth and postpartum blood loss.

We hypothesized that the implementation of PCI would be associated with:

- lower neonatal morbidity and fewer markers of neonatal compromise
- no increase in the cesarean section rate, and
- changes in intrapartum management consistent with a contraction‐reduction strategy; we also prespecified assessment of postpartum blood loss as a potential unintended consequence

## 2 MATERIAL AND METHODS

### 2.1 Study design and setting

We conducted a historical pre–post cohort study at the University Hospital Ulm, a tertiary perinatal center in Germany (~3300 deliveries annually). Two time periods were compared: a pre‐implementation reference period (January 01–December 31, 2018) and a post‐implementation period following full adoption of PCI (May 01, 2022–April 30, 2023). A pre–post design was chosen to minimize contamination during the transition phase.

We recognize that the time gap introduces potential for time‐varying confounding due to secular changes in obstetric and neonatal practice. To mitigate this, we

- restricted the cohort to term singleton pregnancies with intended vaginal birth,
- applied case‐mix adjustment using multivariate models and propensity score matching, and
- prespecified analyses of intrapartum management variables to assess whether observed practice changes were directionally consistent with the implementation strategy.

In addition, leadership positions in the labor ward—both midwifery and medical—remained unchanged across both study periods.

As a supportive context, we also summarized selected routine quality indicators across 2018–2024 (Table 6) to visualize longer‐term trends.

### 2.2 Intervention: PCI implementation

Implementation comprised a multicomponent program comprising:

- An initial 8‐h department‐wide training led by two external experts, comprising all 40 doctors (juniors and seniors) and most of the midwives (43/46) was held in November 2019.
- Weekly fixed‐time online webinars (theory + Q&A; later reduced to fortnightly), weekly in‐person case discussions embedded in routine handover/shift structures for 15 months (January 2020–March 2021).
- A “champions model” (two junior doctors and two midwives) to provide facilitation and bedside support.
- Expanded Morbidity and Mortality conferences with physiology‐focused micro‐teaching.
- A go‐live on May 1, 2022, introducing the PCI sticker as standard documentation with escalation and sign‐off for pathological tracings.
- Sustainment measures including mandatory onboarding (8‐h online theory + structured self‐study) and twice‐yearly refreshers.

Fidelity was monitored by auditing utilization of the physiology‐oriented CTG sticker in routine documentation, maintaining a low‐threshold staff feedback mechanism (“CTG feedback box”), and conducting an internal staff survey on acceptability, usability, and satisfaction.

### 2.3 Participants

Inclusion criteria were singleton pregnancies ≥37 + 0 weeks with intended vaginal birth and cephalic presentation.

Exclusion criteria were insufficient documentation for gestational age, UA pH or mode of delivery, multiples, antepartum intrauterine demise, and planned elective cesarean section.

### 2.4 Variables

We prespecified 19 clinically relevant covariates for adjustment including maternal, pregnancy and intrapartum covariates. Maternal age and pre‐pregnancy body mass index (BMI), gravidity/parity, gestational age, prenatal risk factors and comorbidities (e.g., prior cesarean, hypertensive disorders, diabetes and related medications), labor management (induction, oxytocin, tocolysis, analgesia), key intrapartum factors (e.g., fetal presentation, delivery position, contraction frequency, operative maneuvers), and ultrasound/Doppler parameters (including fetal biometry and amniotic fluid indices).

Suspected intrapartum infection: The operational definition changed after implementation (pre: maternal temperature >38.0°C; post: maternal temperature >38.0°C or CTG features suggestive of inflammatory stress, including sustained baseline rise >10% without decelerations and loss of cycling). This was treated as a time‐varying co‐intervention in the interpretation of intrapartum management outcomes.

### 2.5 Endpoints

The primary outcome was a prespecified composite neonatal morbidity endpoint, defined as admission to NNU plus at least one marker of compromise (umbilical artery pH <7.15, base deficit >16, or 5‐min Apgar score <7). Universal umbilical cord blood sampling of both the umbilical artery and vein is standard practice in our unit. We intentionally selected relatively liberal thresholds to maximize sensitivity for detecting clinically meaningful changes within the available sample size and study period, and because even mild acidemia or low Apgar scores may prompt NNU admission. Moreover, the clinical meaning of a “good” outcome is context‐dependent; from a family‐centerd perspective, early mother–infant separation due to NNU transfer represents an important adverse outcome. Therefore, NNU admission was required as the anchor component of the composite; NNU outcome comprised admissions to the neonatal ward and transfers to the neonatal intensive care unit (NICU); these were analyzed together as a single binary endpoint. We reviewed the obstetric and NNU standard operating procedures across both study periods and identified no changes in the criteria, thresholds, or definitions governing postnatal transfer/admission to the NNU, including transfers to neonatal intensive care.

Secondary neonatal outcomes included umbilical artery pH, base deficit, 5‐min Apgar, and NNU admission. Maternal/intrapartum outcomes included mode of birth, fetal scalp blood sampling, intrapartum use of oxytocin and tocolysis, duration of second stage of labor, and quantified postpartum blood loss. Balancing measures included the cesarean section rate and markers of maternal morbidity. Postpartum blood loss ascertainment: Postpartum blood loss was estimated by the attending midwife and physician using a calibrated collection drape/bag and/or weighing blood‐soaked drapes and pads. Blood loss was recorded in milliliters (mL) in the electronic record. The documentation method was unchanged across study periods.

### 2.6 Statistical analysis

We summarized categorical variables as *n* (%) and continuous variables as mean (standard deviation, SD) or median (interquartile range, IQR) as appropriate. Group comparisons used *χ* ^2^/Fisher's exact test and *t*‐test/Mann–Whitney *U* test given the scale of measurement. Multivariate logistic regression models and propensity‐score‐matched tests estimated adjusted associations between study period and outcomes, reporting adjusted odds ratios (aOR) or mean differences with 95% confidence intervals. Missing data handling (complete‐case) was prespecified. As an additional sensitivity analysis, we conducted propensity score matching analysis to improve comparability between the pre‐ and post‐implementation cohorts and to assess the robustness of the main findings to residual confounding.

Propensity score matching was performed based on a two‐step variable selection. The model includes a prespecified set of clinically relevant maternal, pregnancy and intrapartum covariates (as mentioned above), which were restricted to those with <10% missing data and were additionally retained if they differed between groups and were associated with the composite neonatal outcome and cesarean section rate (variables meeting both criteria were included). We used the same covariate set for propensity‐score matching and logistic regression modeling. Variables considered downstream of intrapartum management and therefore susceptible to post‐treatment bias (e.g., 5‐min Apgar score and postpartum blood loss) were not included in the sensitivity analysis. The covariates that fulfilled the above‐mentioned criteria and were included in the adjusted analyses were maternal BMI, multiparity, and use of tocolysis.

For matched analyses, we report Mantel–Haenszel common odds ratios (common OR) with 95% confidence intervals.

### 2.7 Reporting

The manuscript follows STROBE guidance for observational studies.

An artificial intelligence tool (ChatGPT; OpenAI, large language model) was used solely for English‐language editing and phrasing of the manuscript; no content (data, analyses, results, or conclusions) was generated by AI, and the tool did not fulfill any role as an author.

## 3 RESULTS

### 3.1 Cohort characteristics

**FIGURE 1:**
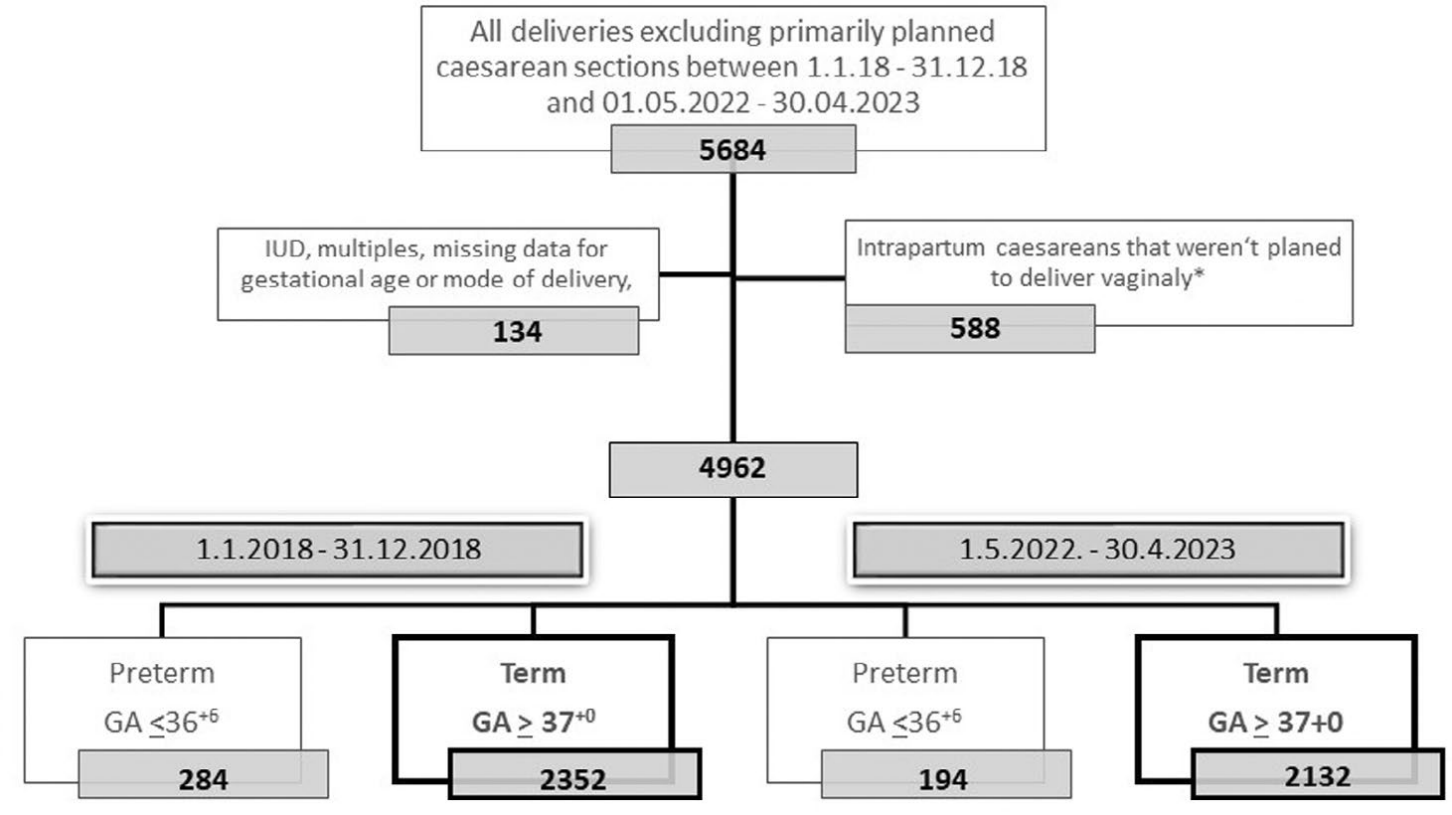
Study flow diagram: Flow diagram of cohort selection for the pre‐implementation (2018) and post‐implementation (2022/23) periods, including inclusion/exclusion criteria and the final analytical cohorts (bold) used for the primary analysis and propensity score‐matched analysis. GA, gestational age; IUD, antepartum intrauterine demise. *Cases in which a cesarean section was planned, but labor had already commenced (e.g., rupture of membranes, contractions). In these cases, cardiotocography (CTG) was performed solely for fetal monitoring from admission to the labor ward until the cesarean section was performed.

A total of 4484 births met the inclusion criteria: 2352 in 2018 and 2132 in 2022/23 (see Figure 1). Baseline maternal and pregnancy characteristics are shown in Table 1. Key differences between the periods included BMI, parity, and hypertensive disorders, which were accounted for in adjusted analyses.

**TABLE 1:** Data are presented as *n* (%) for categorical variables and as median (range) or median (IQR) for continuous variables, as specified in the table.

Baseline maternal and pregnancy characteristics					
Total	2018		2022/23		*p*‐value
	2352		2132		
	Median	Range (min–max)	Median	Range (min–max)	
Maternal age (year)	32	(15–52)	32	(15–48)	0.290
Body mass index	23.5	(16. 2–48.8)	23.8	(15.4–63.3)	0.017
Gestational age	39 + 3	(37 + 0–42 + 0)	39 + 3	(37 + 0–42 + 1)	0.178
Primiparity	1134	48.2%	956	44.8%	0.007
Multiparity^a^	1173	49.9%	1164	54.6%	
Gestational diabetes	334	14.2%	320	15.0%	0.854
Hypertensive disorders	173	7.4%	122	5.7%	0.003

*Note*: *p*‐values compare the pre‐implementation (2018) and post‐implementation (2022/23) cohorts using *χ* ^2^ test or Fisher's exact test for categorical variables and *t* test or Mann–Whitney *U* test for continuous variables, as appropriate. Gestational diabetes was defined as hyperglycemia first recognized during pregnancy not meeting criteria for overt diabetes.

Abbreviations: max, maximum; min, minimum.

^a^ Multiparity includes both history of vaginal and cesarean birth.

Compared with 2018, the 2022/2023 cohort showed a marked increase in intrapartum tocolysis (35.65% vs 16.88%, *p* < 0.001; bolus tocolysis 16.89% vs 7.14%, *p* < 0.001) and a reduction in intrapartum oxytocin use (32.97% vs 39.03%, *p* = 0.0025). Mean duration of labor was longer (8:46 vs 7:37 h, *p* < 0.001), while fetal scalp blood sampling decreased substantially (1.92% vs 4.97%, *p* < 0.001). The post‐implementation group showed a statistically significant reduction in intrapartum interventions, including episiotomy (6.8% vs 3.45%, *p* < 0.001) and fetal blood sampling (4.97% vs 1.92%, *p* < 0.001). Maternal fever was identified more often after implementation of PCI but did not reach significance (7.74% vs 6.89%, *p* = 0.064). Maternal blood loss increased, with higher average blood loss (497 vs 438 mL, *p* < 0.001); the raw data showed that cesarean section rates were higher (13.27% vs 10.25%, *p* = 0.003), whereas emergency cesarean sections did not differ significantly (*p* = 0.765) (Table 2).

**TABLE 2:** Intrapartum characteristics.

Intrapartum characteristics			
Total	2018	2022/2023	*p*‐value
	2352	2132	
Intrapartum tocolysis	397 (16.88%)	760 (35.65%)	<0.001
Bolus tocolysis	186 (7.14%)	360 (16.89%)	<0.001
Intrapartum oxytocin	918 (39.03%)	703 (32.97%)	0.0025
Duration of labor (mean)	7:37 h (SD ± 6:45 h)	8:46 h (SD ± 7:31 h)	<0.001
Fever^a^	162 (6.89%)	165 (7.74%)	0.064
Malpresentation	201 (8.55%)	116 (5.44%)	<0.001
Episiotomy	160 (6.8%)	74 (3.45%)	<0.001
Spontaneous vaginal delivery	1882 (80.02)	1632 (76.55%)	0.003
Ventouse delivery	229 (9.74%)	217 (10.18)	
Cesarean section	241 (10.25%)	283 (13.27%)	
Emergency cesarean section	13 (0.55%)	18 (0.84%)	0.765
FBS	117 (4.97%)	41 (1.92%)	<0.001
Median blood loss (standard deviation)	438 mL (SD ± 290 mL)	497 mL (SD ± 364 mL)	<0.001

*Note*: Before: Mat. temperature >38.0°; After: Maternal temp. >38.0°C or rise in BL >10% without decelerations and loss of cycling. Data are presented as *n* (%) unless otherwise specified. “Duration of labor” refers to the time from onset of active first stage to delivery. Blood loss was estimated by the attending midwife and physician using a calibrated collection drape/bag and/or gravimetric assessment (weighing blood − soaked drapes/pads) and recorded in milliliters (mL). Cesarean sections include all intrapartum cesarean sections that were not done as an emergency by national definition. Emergency cesarean sections need to have a decision‐to‐delivery‐time frame of less that 20 min and follow a prespecified algorithm. *p*‐values compare the two study periods using *χ* ^2^/Fisher's exact test, *t* test, or Mann–Whitney *U* test, as appropriate.

Abbreviations: CTG, cardiotocography; Duration of labor, time of active first stage until delivery; FBS, fetal blood sampling; SD, standard deviation.

^a^ Definitions for fever changed after implementation of PCI.

### 3.2 Primary outcome

The composite neonatal morbidity endpoint occurred in 2.44% after versus 3.61% before implementation of PCI (*p* = 0.024, OR 0.667). Likewise, there was a significant decrease in neonatal acidosis for pH <7.15 and pH <7.10. Transfer rate to NNU declined significantly from 13.18% to 9.19% (OR 0.667, *p* < 0.001) (Table 3).

**TABLE 3:** Neonatal outcome parameter before and after implementation: OR with 95% confidence intervals.

Neonatal outcome parameters				
Total	2018	2022/2023	Odds ratio	*p*‐value
	2352	2132	95%‐confidence‐interval	
pH <7.00	13 (0.55%)	9 (0.42%)	0.763 (0.287–1.933)	*p* = 0.670
pH <7.10	91 (3.87%)	54 (2.53%)	0.646 (0.450–0.949)	*p* = 0.014
pH <7.15	273 (11.61%)	193 (9.05%)	0.758 (0.621–0.925)	*p* = 0.005
Transfer to NNU	310 (13.18%)	196 (9.19%)	0.666 (0.548–0.808)	*p* < 0.001
Composite NN‐morbidity	85 (3.61%)	52 (2.44%)	0.667 (0.460–0.958)	*p* = 0.024

*Note*: The primary composite neonatal morbidity endpoint was defined as admission/transfer to the neonatal unit (NNU) plus at least one marker of compromise (umbilical artery pH <7.15, base deficit >16, or 5‐min Apgar score <7). NNU admission includes admissions to the neonatal ward and transfers to neonatal intensive care (NICU). Odds ratios are reported for post‐implementation (2022/23) versus pre‐implementation (2018). Fisher's exact test *p*‐values are two‐sided.

Abbreviations: NN, neonatal; NNU, neonatal unit.

The study group was independently associated with reduced odds of the composite neonatal outcome (aOR 0.605, 95% CI 0.415–0.872; *p* = 0.008), with no multicollinearity detected (Table 4).

**TABLE 4:** Multivariate analysis for composite neonatal outcome: Data are presented as adjusted odds ratios (aORs) with 95% confidence intervals (CIs) from a multivariate logistic regression model for the composite neonatal outcome.

Multivariate analysis for composite neonatal outcome			
Variable	aOR	Confidence interval	*p*‐values
Study group (2022/23)	0.605	0.415–0.872	0.008
Tocolysis	1.899	1.275–2.791	0.001
Multiparity^a^	0.663	0.508–0.842	0.001

*Note*: The reference group is births in 2018; the study group is births in 2022/2023. Maternal weight was modeled as a continuous variable.

^a^ Multiparity was defined as any previous birth (vaginal and/or cesarean). Covariates were included if clinically relevant, differed between groups, and were associated with the outcome; collinearity was assessed, and variables on the causal pathway were excluded to exclude posttreatment bias.

Propensity score matching yielded 1639 matched pairs (from initial samples of 2325 and 2132 participants), reducing the maximum standardized mean difference from 0.177 to 0.006. The association remained robust, with the study group showing lower odds of an adverse composite neonatal outcome (common OR 0.54, 95% CI 0.354–0.836; *p* = 0.0039).

Multiparity was more frequent in the study group. This residual imbalance was small and unlikely to meaningfully bias the matching, as the effect of tocolysis dominated the multivariate model. A Firth‐penalized logistic regression confirmed the robustness of the results, yielding effect estimates consistent with the primary analysis.

### 3.3 Maternal and intrapartum outcomes

Before matching, we fitted a univariate logistic regression model to estimate the crude before‐after group effect on cesarean delivery, which indicated a significantly higher cesarean rate in the post‐intervention group. In contrast, this apparent increase was no longer observed in the multivariate model or after propensity score matching adjusting for pre‐and intrapartum covariates. The cesarean rate remained unchanged, with a nonsignificant trend toward a lower rate in the post‐intervention group (OR 0.858, CI 0.722–1.071) (Table 5).

**TABLE 5:** Multivariate analysis for cesarean section rate.

Multivariate analysis for cesarean section rate			
Variable	OR	95%‐confidence interval	*p*‐value
Study group (2022/23)	0.858	0.690–1.066	0.169
Tocolysis^b^	7.257	5.832–9.049	<0.001
Maternal BMI	1.048	1.031–1.065	<0.001
Multiparity^a^	0.631	0.540–0.730	<0.001

*Note*: Multivariate logistic regression was used to estimate adjusted odds ratios (aOR) with 95% confidence intervals for cesarean section. The exposure variable “study period” compares post‐implementation (2022/23) with pre‐implementation (2018). Collinearity was assessed using variance inflation factors (VIF), with values <5 indicating low collinearity.

Abbreviation: OR, odds ratio.

^a^ Multiparity includes both history of vaginal and cesarean birth.

^b^ Tocolysis remained a strong predictor; robustness was confirmed using Firth‐penalized logistic regression.

Use of tocolysis and oxytocin was changed in line with physiological management principles (Table 2). Postpartum blood loss was significantly higher post‐implementation (438 vs 497 mL).

## 4 DISCUSSION

This unit‐wide implementation of PCI (including structured training, bedside facilitation and documentation changes) was associated with lower neonatal morbidity and fewer NNU admissions in term births. Importantly, these improvements were observed without an increase in cesarean section rates after case‐mix adjustment. Postpartum blood loss was higher in the post‐implementation period. While this difference reached statistical significance, the observed mean difference of approximately 60 mL is unlikely to be clinically meaningful in isolation, and its clinical relevance remains uncertain. The finding should therefore be interpreted with caution. More clinically meaningful categorical bleeding outcomes, such as blood loss ≥1000 mL, would have been preferable, but were not available in the prespecified outcome format for this analysis. Routine postpartum oxytocin administration was not standard practice in our unit at the time, and protocols have since been adjusted accordingly. Unfortunately, maternal transfers to the Intensive Care Unit were not recorded. No maternal deaths occurred within the observed time frames.

We observed a significant prolongation of labor duration in the post‐implementation period. This may be partly attributable to the increased use of tocolysis. However, it likely also reflects contemporaneous changes in intrapartum management, as the post‐implementation period took place after the publication of the S3‐Guideline on vaginal birth at term, which includes more liberal time‐limit targets during labor.^13^ Therefore, this may have influenced clinical decision‐making in our unit.

In addition, the timing of intrapartum cesarean delivery differed between periods. In 2022/23, cesarean sections were performed significantly later in labor, with a higher median cervical dilatation at cesarean compared with 2018 (8 vs 6 cm, *p* < 0.001).

The observed association between tocolysis and intrapartum cesarean delivery likely reflects confounding by indication. In our practice, tocolysis was most often administered for suspected acute fetal compromise, frequently in the context of uterine tachysystole, as an intrauterine resuscitative measure. Cesarean delivery was undertaken when fetal status did not subsequently improve. We cannot quantify how many cesarean sections may have been avoided following successful tocolysis, but within a physiology‐based approach, its use should be viewed as a response to an identified fetal problem rather than a modifiable “risk factor.” Therefore, avoiding tocolysis with the aim of reducing cesarean rates would be inappropriate and could jeopardize fetal safety.

A statistically significant reduction was also observed in two intrapartum interventions, namely episiotomy and fetal blood sampling, in the post‐implementation group. This is likely to be clinically relevant, as fewer episiotomies may reduce maternal morbidity (pain and recovery time), while lower use of fetal blood sampling is consistent with the limited evidence supporting its benefit and with current NICE guidance, which no longer recommends its routine use.^12^, ^14^, ^15^ A physiology‐based framework may have improved clinicians' confidence in distinguishing compensatory fetal responses from true deterioration, thereby potentially reducing urgent interventions triggered by pattern‐based overinterpretation; however, given the observational pre–post design, causality cannot be inferred.

Implementation fidelity was monitored through the review of CTG sticker utilization, establishment of a low‐threshold feedback mechanism (“CTG feedback box”) to allow staff to raise concerns or request discussion, and an internal staff survey assessing acceptability, usability, and satisfaction with the approach was performed.^16^

Current evidence from the medical literature does not provide clear proof that the implementation of the FIGO‐CTG‐Score has improved neonatal outcomes. Multiple primary and review articles indicate that the introduction of the FIGO 2015 classification has improved interobserver agreement for normal CTG findings; however, compared with earlier classification systems (e.g., FIGO 1987), it appears to be associated with reduced sensitivity for detecting pathological CTG patterns and for identifying fetal acidemia.^17^, ^18^, ^19^, ^20^

This evaluation contributes to the literature by linking an interpretive framework to real‐world clinical decision‐making and observed outcomes. Many comparative studies of CTG interpretation systems rely on historical trace re‐classification, in which the same recordings are scored under different frameworks by independent reviewers.^3^, ^21^, ^22^, ^23^ While these designs are informative for agreement and construct validity, they do not capture effectiveness when embedded in routine care and management responses. By analyzing outcomes following unit‐wide implementation and using case‐mix adjustment, our study provides pragmatic evidence on the association between physiological interpretation and neonatal outcomes in clinical practice.

The observed pattern of improved neonatal outcomes without an increase in operative births suggests that a physiology‐based interpretation may help distinguish between fetuses requiring urgent delivery and those who may benefit from conservative, contraction‐reducing measures. This is consistent with emerging evidence from other centers suggesting that implementation of PCI may also be associated with improvements in maternal and perinatal outcomes.^24^, ^25^, ^26^, ^27^

The direction of these findings is consistent with the recent NICE guidance on intrapartum fetal monitoring, which incorporates key elements aligned with physiological CTG principles into its interpretation framework^12^; however, given the observational pre–post design, the results should be interpreted as association rather than definitive evidence of causality.

If confirmed by multicenter studies, PCI could represent a pragmatic, unit‐wide strategy to reduce potentially preventable neonatal compromise while maintaining operative birth rates. The observed increase in postpartum blood loss emphasizes the need to monitor maternal safety outcomes and refine protocols when adopting physiology‐based intrapartum management. However, further multicenter comparative studies are needed to fully explore the effects of this emerging CTG interpretation approach.

Strengths of this study include a large clinical cohort from a high‐volume tertiary center and comprehensive outcome ascertainment. The pre–post design represents a real‐world evaluation of routine clinical practice, providing pragmatic evidence of the effects of PCI. However, limitations include the non‐randomized nature of the pre–post design, which is susceptible to secular trends and unmeasured confounding. While we adjusted for confounders using propensity score matching, residual confounding cannot be completely excluded, and the findings should be interpreted as associations rather than causal effects. However, NNU admission criteria or management protocols did not change during the study period.

To mitigate the risk of bias, we also reviewed our hospital quality assurance data from 2018 to 2024, which demonstrate a continuous decline in acidosis (pH <7.0, <7.10, <7.20) and a steady reduction in transfers to the NNU (Table 6). Although these data are not directly transferable to our study cohort (due to differences in gestational age, multiple pregnancies, and planned cesarean sections), they provide supportive evidence for the positive trend in neonatal outcomes following implementation of PCI. Future studies should consider interrupted time series analysis using routinely collected data to further separate the effect of implementation from temporal changes in obstetric and neonatal practice.

**TABLE 6:** Quality assurance data.

Parameters	2018	2019	2020	2021	2022	2023	2024
Number of deliveries	3169	3237	3161	3250	2992	3174	3159
pH <7.0 (in %)^a^	0.58	0.59	0.49	0.36	0.29	0.34	0.37
pH 7.00–7.09 (in %)^a^	2.51	2.99	2.46	1.97	2.3	1.65	1.81
pH 7.10–7.19 (in %)^a^	20.41	19.59	17.77	16.67	18.29	16.16	15.89
pH >7.20 (in %)^a^	75.65	76.14	78.52	79.21	77.75	81.12	81.17
5 min APGAR 0–6 (in %)^a^	1.42	1.6	1.58	2.18	2.11	1.89	1.87
5 min Apgar >7 (in %)^a^	97.51	97.07	96.28	95.7	96.47	96.58	98.99
Transfer rate to NNU (in %)^a^	22.74	22.91	19.11	16.85	17.81	18.42	17.42
Observed/expected (O/E) rate of acidosis (pH <7.0) in our unit^b^	OR 2.14 (CI: 1.25–3.66)	1.92 (CI: 1.12–3.28)	1.74 (CI: 0.97–3.11)	1.55 (CI: 0.84–2.85)	1.25 (CI: 0.63–2.45)	0.91 (CI: 0.42–1.98)	1.05 (CI: 0.51–2.15)
Observed/expected (O/E) rate of acidosis (pH <7.0) in term singletons nationwide^b^	OR 1.07 (CI: 1.01–1.12)	1.01 (CI: 0.96–106)	1.00 (CI: 0.95–1.05)	0.96 (CI: 0.91–1.01)	0.97 (CI: 0.92–1.02)	1.04 (CI: 0.99–1.10)	1.04 (CI: 0.99–1.10)

Abbreviation: CI, confidence interval.

^a^ Including all deliveries in our unit (multiples and singeltons, Preterms and term deliveries as well as planned cesarean sections).

^b^ All live‐born term singletons (37 + 0 to 42 + 0 weeks' gestation) with an umbilical arterial cord blood pH.

Moreover, the intervention was a multicomponent implementation strategy, which included the interpretive framework, training, bedside facilitation, and revised documentation. This multilayered approach makes it difficult to attribute observed outcomes solely to the PCI framework itself. The improvements observed are more likely to reflect the combined effect of the full implementation strategy. The independent contribution of each component (training vs interpretation method) cannot be definitively disentangled in this study.

Additionally, recent findings show that CTG training in Germany has considerable potential for improvement (unpublished observations, currently under review). Changes to training strategies were implemented in our unit alongside the introduction of the PCI framework. While a substantial increase in training occurred during the study period, it remains unclear to what extent the observed changes in neonatal outcomes are attributable to the increased training versus the PCI approach itself.

Finally, although certain management aspects of PCI are not fully aligned with the current German guideline framework, emerging expert guidance and the NICE intrapartum fetal monitoring guideline support key underlying principles of this approach, particularly contextualized interpretation beyond CTG pattern recognition alone.^12^ In this context, our findings may support further evaluation of physiology‐based intrapartum management in other maternity settings.

## 5 CONCLUSION

The implementation of PCI was associated with improved neonatal outcomes without increasing cesarean sections, but with higher postpartum blood loss. Further multicenter evaluations using quasi‐experimental designs are needed to confirm its effectiveness, understand the underlying mechanisms, and optimize maternal safety.

## AUTHOR CONTRIBUTIONS

SA: Implementation, clinical execution, coordination of data collection, data interpretation, draft. HS: implementation, draft. KS: statistical analysis. AS: Review, editing MD: clinical execution, correction of draft, Data curation. WJ: correction of draft. BH: clinical execution and feedback, correction of draft. FR: Implementation, clinical execution, supervisor, data interpretation, and correction of draft.

## FUNDING INFORMATION

No funding was received to conduct this study.

## CONFLICT OF INTEREST STATEMENT

SA and FR report roles as scientific leads and lecturers for the CTG Xpert courses in Germany. SA and FR have received speaker honoraria for teaching on physiological CTG interpretation and SA has received remuneration for authoring a CTG‐related chapter for the AMBOSS learning platform. All other authors declare no competing interests.

## ETHICS STATEMENT

The study was approved by the Ethics Committee of the University of Ulm (Approval No. 506_21), and the corresponding amendment was granted on January 15, 2024.

## Data Availability

The data that support the findings of this study are available on request from the corresponding author. The data are not publicly available due to privacy or ethical restrictions.
